# Novel immunochromatographic test for rapid detection of anti-factor H autoantibodies with an assessment of its clinical relevance

**DOI:** 10.3389/fimmu.2024.1527016

**Published:** 2025-01-24

**Authors:** Santiago Rodríguez de Córdoba, Andrea Reparaz, Daniel Sanchez, Sheila Pinto, Lucia Juana Lopez, Héctor Martin Merinero, Iria Calvete, Julian Perez-Perez, Sydney S. Jellison, Yuzhou Zhang, Richard J. H. Smith, Inmaculada Moreno, Mercedes Dominguez

**Affiliations:** ^1^ Centro de Investigaciones Biológicas Margarita Salas, Consejo Superior Investigaciones Científicas (CSIC), Madrid, Spain; ^2^ Secugen S. L., Madrid, Spain; ^3^ Molecular Otolaryngology and Renal Research Laboratories, Carver College of Medicine, University of Iowa, Iowa City, IA, United States; ^4^ Unidad de Inmunología Microbiana, Centro Nacional de Microbiología, Instituto de Salud Carlos III, Madrid, Spain

**Keywords:** anti factor H autoantibodies, immunochromatographic test, complement, atypical hemolytic uremic syndrome, C3-glomerulopathy, diagnostic test

## Abstract

Factor H (FH) is a crucial complement regulator that prevents complement-mediated injury to healthy cells and tissues. This regulatory function can be disrupted by Factor H autoantibodies (FHAA), which then leads to diseases such as atypical hemolytic uremic syndrome (aHUS) and C3 Glomerulopathy (C3G). In pediatric aHUS, the FHAA incidence is ~10-15%, although in the Indian population, it rises to ~50%. The specific regions of FH targeted by FHAAs correlate with the pathogenic mechanism of the associated disease. In aHUS, FHAAs target the C-terminus, thereby impacting FH ability to recognize cell surfaces. In C3G, in contrast, FHAAs often target the N-terminus, generating an acquired functional FH deficiency. Detection and monitoring FHAAs are decisive for effectively treating patients. Current FHAA analysis normally identify free FHAAs that bind surface-bound FH using ELISA techniques. These methods require well-equipped laboratories and qualified staff, and do not measure FH-FHAA complexes, which can make it difficult to correlate titers with clinical outcomes. The visually-based immunochromatographic test (ICT) described herein allows for quick detection and quantification of IgG and IgM FH-FHAA complexes in human EDTA-plasma or serum. This ICT offers improved detection of FHAAs compared to ELISA as demonstrated by cases where the ICT identifies FH-FHAA complexes in samples that tested negative with the free FHAA ELISA. Importantly, the ICT indirectly informs on the amount of FH that is complexed with FHAAs, thus assessing the significance of the FHAA in disrupting the regulatory function of FH. Overall, this novel assay offers a simple, fast, cost-effective, and, likely, more clinically relevant alternative for diagnosing FHAAs in at-risk populations.

## 1 Introduction

FH autoantibodies (FHAAs) are antibodies that specifically target factor H (FH), an essential regulator of the complement system (1). The complement system is an integral part of the immune system that plays a critical role in identifying and eliminating pathogens, such as bacteria and viruses. FH is vital for controlling complement activity to avoid harm to healthy cells and tissues (2). FHAAs abrogate this control, which leads to the complement dysregulation that is characteristic of specific diseases such as atypical hemolytic uremic syndrome (aHUS; also known as complement-mediated thrombotic microangiopathy or cm-TMA) and C3 Glomerulopathy (C3G) (1, 3–8). In aHUS, for example, FHAAs are identified in ~10-15% of pediatric cases, although in certain ethnic groups, such as those in India, the prevalence reaches ~50% of children with aHUS (9, 10). Strikingly, FHAAs in aHUS are most often associated with homozygosity for the tandem deletion of the *CFHR*3 and *CFHR1* genes (∆*CFHR3-CFHR1*) (11–13). In adults, the presence of FHAAs is markedly lower and is at times indicative of an underlying monoclonal gammopathy (3). FHAAs can be IgG, IgM or a combination of both (14, 15).

FHAAs contribute to disease by inhibiting critical functions of FH (16, 17). In the context of pathologies involving FHAAs, there is a distinction in the binding regions of these autoantibodies to FH that is disease specific. In aHUS, FHAAs primarily target the C-terminal short consensus repeats (SCRs), thereby interfering with the regulatory functions of FH on cell surfaces, which is critical to prevent complement-mediated damage. SCR19-20 domains are identified as the main binding sites in these cases. Conversely, in diseases like C3G, the autoantibodies predominantly bind to the N-terminal SCRs of FH (7, 16, 18). This region is crucial for the regulatory roles of FH in the fluid phase, such as decay-accelerating activity and cofactor activity, which are essential for controlling the complement cascade. The differences in FHAA binding sites reflect the pathophysiological variations between these diseases and underscore the complexity and specificity of immune responses in complement-mediated disorders. This specificity not only impacts the clinical presentation of disease but also influences the diagnostic approach and potential therapeutic strategies for each condition. The detection and monitoring of FHAAs are clinically important for the effective treatment of patients (3).

Circulating FH-FHAA complexes have been shown to correlate more accurately with disease activity than free FHAA titers alone (16). Assays for detecting these complexes have been described in several studies (10, 16); however, most clinical laboratories employ ELISA techniques to identify only free FHAAs using techniques that involve attaching purified FH to a surface (1, 19). Antibody titers are generally expressed in Arbitrary Units (AU), typically referenced to a dilution of a standard serum with a high titer of FHAA. Importantly, ELISA methods may overestimate the binding affinity of FHAAs to FH and without data on FHAA complexes, correlating levels of free FHAAs with clinical outcome can be challenging. In addition, these ELISA techniques require both specialized settings and expert personnel, which are not available in all parts of the world. Sending samples to reference laboratories also incurs costs and significant delays in implementing therapies for patients. As such, there is an urgent need to develop new assays that are cheaper and easier to perform for diagnosing FHAAs in at-risk populations.

The immunochromatographic test (ICT) described herein is the first visually based method to detect FHAAs of the IgG and IgM types and quantify the levels of FH-FHAA complexes in human serum or EDTA-plasma. Additionally, the assay distinguishes between FHAAs targeted to different regions of FH.

## 2 Materials and methods

### 2.1 Generation of immunochromatographic cassettes

**Figure 1:**
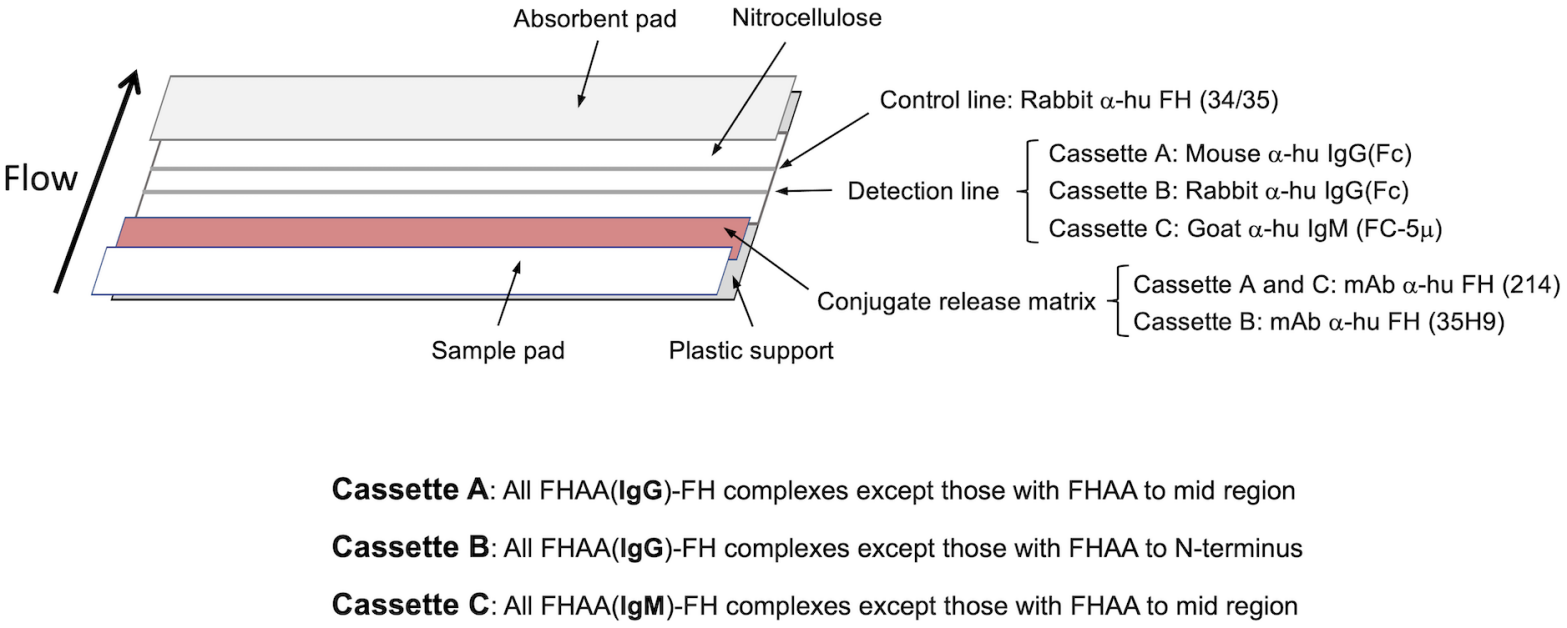
Design of the three cassettes to make the immunochromatographic test (ICT). Antibodies used in the different parts of the three immunochromatographic strips are indicated and their detection capabilities shown beneath (See also Materials and Methods).

We developed three immunochromatographic cassettes to detect all types of complexes formed between human FH and FHAA (**Figure 1**).

#### 2.1.1 Cassette A

This cassette is designed to detect complexes of human FH with FHAAs of the IgG type directed to the N- and/or C-terminal regions of FH. Cassette A will not detect FHAAs directed to mid-region (surrounding SCR14) of FH. To build cassette A, an affinity purified mouse polyclonal antibody anti-human IgG specific for the γ chain (made in house; Detection line) and a rabbit anti-human FH polyclonal antibody (34/35 (made in house); Control line) are coated onto a laminated nitrocellulose membrane type 200CNNPH-N-SS60-L2-P25 from Advanced Microdevices (Haryana, India) using an Easy Printer model LPM-02 from Advanced Microdevices (Haryana, India). A mouse monoclonal antibody (214; made in house) directed to SCR-11 of human FH is conjugated to 40nm colloidal gold nanoparticles from BBI solutions (Crumlin, UK), as described in Parolo et al. (20). The antibody-coated gold nanoparticles are then deposited onto a conjugate release matrix type PT-R7 from Advanced Microdevices (Haryana, India). The printed laminated nitrocellulose membrane, the air-dried conjugate release matrix carrying the gold nanoparticles, a sample pad type GFB-R7L (0.6) and an absorbent pad type AP-110, both from Advanced Microdevices (Haryana, India) are then assembled as shown in **Figure 1** and cut into 4.5mm strips, which are placed inside a plastic rapid test cassette type DEVICE-3 from Advanced Microdevices (Haryana, India).

For developing the assay and to minimize the hook effect characteristic of this type of ICT, plasma-EDTA is diluted at a 1:40 ratio in TTBSA buffer (50mM Tris pH 7.4, 150mM NaCl, 0.2% Tween20, 1% BSA) at room temperature. 50μL of this diluted sample are applied into the sample hole on the rapid test cassette. Three minutes after the application, 50μL of TTBSA buffer are added into the sample hole on the cassette and the test is allowed to develop fully for another 15-20 minutes. The results of the test are read with a Colloidal Gold Rapid Test Strip Reader CHL-TSR100 from Guangzhou Iclear Healthcare Limited (Guangzhou, China) within 10-20 minutes after the development process is complete.

The gold-labeled anti-FH monoclonal antibody binds both to FH and any IgG:FH (FHAAs:FH) that are present. A control color line that identifies FH bound to the gold-labeled anti-FH monoclonal antibody appears in the control line and serves as an internal control indicating that the immunochromatographic process is normal. The IgG:FH–gold-labeled anti-FH monoclonal complexes, if present, move along the test strip and bind to the anti-human IgG antibody in the detection line of the nitrocellulose membrane. This creates an immobilized “anti-human IgG antibody–IgG:FH–gold-labeled anti-FH antibody” sandwich complex, which turns red. If there are no IgG:FH complexes in the sample, no sandwich complexes form in the detection line. The color intensity of the detection line is proportional to the concentration of IgG:FH complexes in the sample (see also Interpretation of the Results).

#### 2.1.2 Cassette B

This cassette detects complexes of human FH with FHAA of the IgG type that are not directed to the N-terminal region of human FH. As compared to Cassette A, Cassette B uses gold nanoparticles coated with a different mouse monoclonal antibody (35H9; made in house) that is directed to the N-terminal region of human FH, and a rabbit polyclonal antibody anti-human IgG specific for the γ chain (Thermo Fisher Scientific) in the detection line (as opposed to a mouse polyclonal antibody anti-human IgG specific for the γ chain in Cassette A). Cassettes A and B provide similar results in the detection area, unless the FHAAs are directed to the N-terminal or mid region of human FH and then no sandwich complexes, or very much decreased amounts of these complexes, are formed in the detection line of cassette B or cassette A, respectively (see also Interpretation of the Results). Development of the assay in cassette B is otherwise identical to that with cassette A.

#### 2.1.3 Cassette C

This cassette detects complexes of human FH with FHAA of the IgM type directed to the N-terminal and C-terminal regions of human FH. Cassette C will not detect FHAAs directed to mid-region (surrounding SCR14) of FH. As compared to Cassette A, Cassette C uses a rabbit polyclonal antibody anti-human IgM specific for the µ chain from Jackson Immunoresearch Europe (Ely, UK), which is coated onto the detection line of the nitrocellulose membrane. Sandwich complexes form in the detection line of Cassette C only if the FHAAs are IgM. For Cassette C, plasma-EDTA is diluted at a 1:5 ratio in TTBSA buffer because the hook effect is reduced due to the lower plasma concentration of the IgM; otherwise, the development of the assay is identical to that with Cassette A and Cassette B.

### 2.2 Interpretation of results obtained with the immunochromatographic cassettes

**Figure 2:**
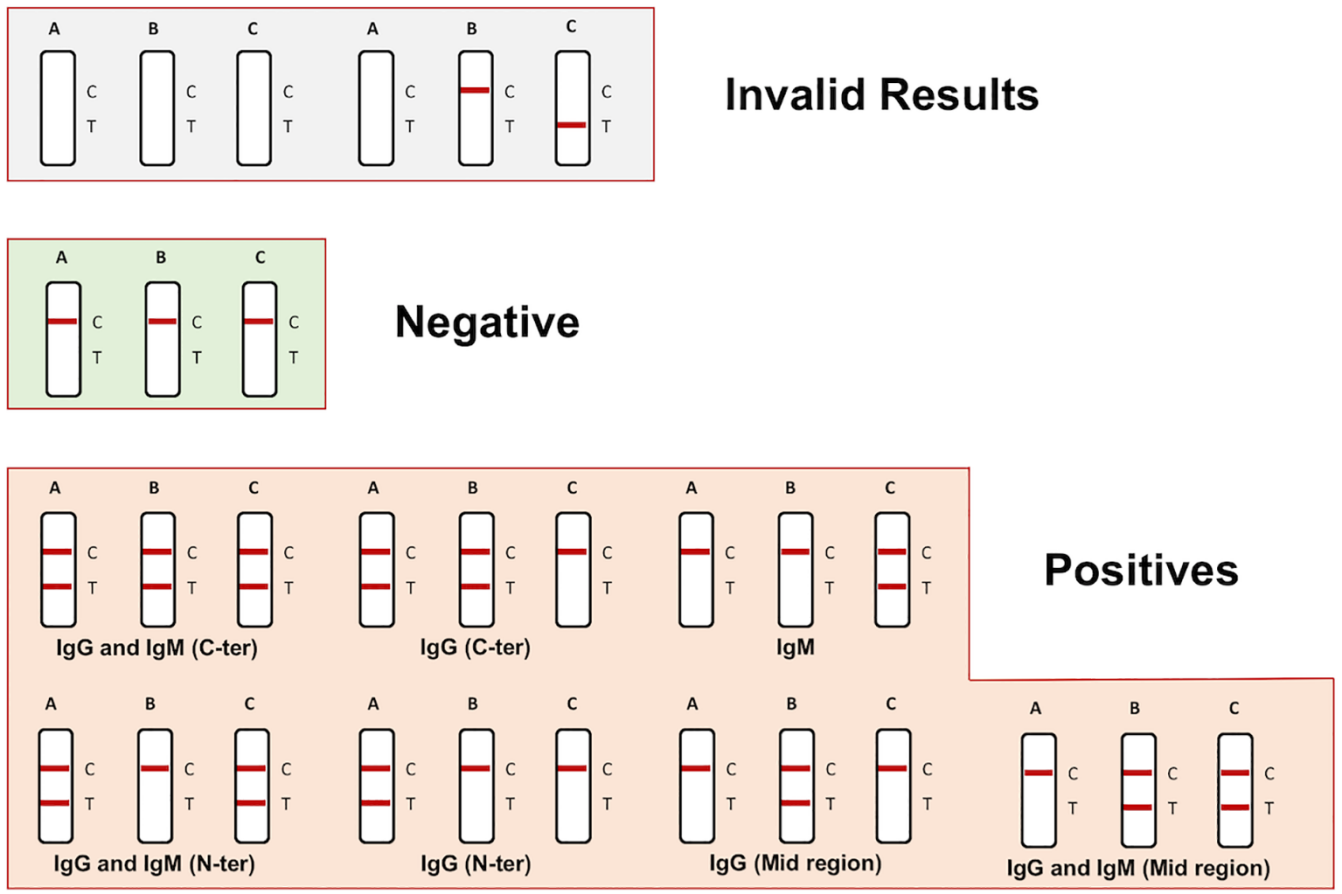
Schematic representation of possible results using the ICT cassettes and their interpretation.

1. Once the development of the ICT is completed, first read the color line in control line “C” to determine whether the results are valid. If no color has developed in the control line or if color appears only at the detection line “T” in one or more cassettes, the test results are invalid (**Figure 2**).
2. If color appears only at the control line “C” in the three cassettes, the ICT result is a valid negative. No FHAAs (IgG and IgM type) bound to FH are present (**Figure 2**).
3. If both detection line “T” and control line “C” appear in one or more cassettes the ICT result is valid positive. The sample is positive for FHAAs and the intensity of the detection line is proportional to the concentration of Immunoglobulin-FH complexes (FHAA-FH complexes). The concentration can be estimated by visual inspection or by scanning the cassette in a densitometric device, as described above. The different combinations of colored detection lines in the three cassettes informs the type of immunoglobulin and FH region targeted by the FHAAs as described in **Figure 2**.
4. The relative intensity of the detection line “T” in cassettes A and B is indicative of the location of the epitope targeted by the FHAA. Thus, while similar intensities in cassette A and B associate with FHAA targeted to the C-terminus (SCR18-20) of FH, a negative or a very reduced intensity in cassette A compared to cassette B indicates the presence of FHAA directed to the mid-region (surrounding SCR14) of FH. And, similarly, a negative or a very reduced intensity in cassette B compared to cassette A indicates the presence of FHAA directed to the N-terminus (SCR1-5) of FH.
5. The intensity at the control line “C” in the cassettes may be significant weaker in samples with high FHAA titers. In these cases, most of FH may be present in the FHAA-FH complexes and the majority of nanoparticles are retained in the detection line “T”.
6. Using samples from healthy control individuals as a reference for negative samples and a Colloidal Gold Rapid Test Strip Reader CHL-TSR100, we considered weak positive samples for FHAA values between 100-200 AU and clearly positive or strong positive samples for FHAA values >200AU. Results can also be easily assessed visually.

### 2.3 ELISA method for detecting free IgG FHAA

FH autoantibodies (FHAAs) are detected as previously described (1, 19), with slight modifications. Briefly, 100 µL of in-house prepared purified human FH at a concentration of 5 µg/mL in Phosphate Buffered Saline (PBS) is added to each well of a 96-well microtiter plate (Costar; high-binding capacity) and incubated overnight at 4°C. After washing three times with TTBSA buffer, free reactive sites are blocked using TTBSA buffer containing 3% BSA for 1 hour at room temperature. A separate blank plate, devoid of FH, is also blocked with the TTBSA buffer containing 3% BSA for 1 hour at room temperature. Patient samples are then added at 1:100 and 1:500 dilutions in TTBSA buffer to both the FH-coated and blank plates, followed by a 1-hour incubation at room temperature. After the incubation, plates are washed extensively in TTBSA containing 1% BSA and then incubated for an additional 30 minutes at room temperature with horseradish peroxidase-labeled goat anti-human IgG antibody (Thermo Fisher Scientific). Post-incubation, plates are washed again, and the enzymatic activity is assessed using o-phenylenediamine dihydrochloride (OPD), with the absorbance measured at 490 nm. To determine titer, the absorbance values from the blank plate are subtracted from those of the FH plate and fitted to a standard curve generated by serial dilutions of a positive control sample, which is assigned an arbitrary value of 35 units (AU) at a 1:1000 dilution in TTBSA. The FHAA titers, expressed in AU, are then normalized based on the expected and observed FHAAs for two positive control samples included in the run.

The large majority of tested controls in our assay were AU=0, with very few exceptions between 0 and 100AU. Samples with values above 100AU were considered positive, samples with 0>AU<100 were undefined, and samples with AU=0 were negative.

### 2.4 Patient and healthy controls samples

#### 2.4.1 Testing and validation cohort IgG-FHAA (Madrid)

The cohort includes 784 patients (both adults and pediatrics) with a diagnosis of aHUS and 325 patients with a diagnosis of C3G who were tested for the presence of IgG FHAAs at the Centro de Investigaciones Biológicas Margarita Salas in Madrid, using the ELISA method. In aggregate, 34 aHUS and 7 C3G patients were positive for FHAAs (AU>100); 28 aHUS and 16 C3G patients were undefined (AU values between 0 and 100); and 722 aHUS and 302 C3G were negative (AU=0). Amongst the negative, 51 aHUS patients and 9 C3G patients were homozygous for the tandem deletion of *CFHR3* and *CFHR1* (∆*CFHR3-CFHR1*). In this ELISA-based cohort, we did not perform epitope mapping and we did not test for FHAAs of the IgM type.

#### 2.4.2 Validation samples for IgM-FHAA and epitope mapping

Validation of the epitope mapping data obtained with the ICT was conducted on 31 samples from the aHUS and C3G cohorts in the Molecular Otolaryngology and Renal Research Laboratories at the University of Iowa. These samples tested positive for IgG FHAA by ELISA (Samples > are considered positive in Iowa) and had epitope mapping determined as previously described (8). Five IgM FHAA-positive samples from the same aHUS and C3G cohorts at the University of Iowa were also used to validate the capacity of the ICT to detect IgM FHAA. The results of the test were read in Iowa with a ESEQuant Flex equipment from DIALUNOX GmbH, Germany. Values above 12AU were considered positive.

#### 2.4.3 Healthy controls

Forty samples from normal healthy individuals collected at the Centro de Investigaciones Biológicas Margarita Salas in Madrid, were also included in the evaluation of the ICT. These samples were useful to determine the positive cut off at 100AU when using the Colloidal Gold Rapid Test Strip Reader.

#### 2.4.4 Clinical data from patients with conflicting ELISA and ICT FHAA results

GN500 is a 55-year-old man with normal kidney function until May 2023. In January 2024, he had a creatinine level of 1.5 mg/mL and proteinuria of 3.4 g/24h, associated with an IgG-Kappa multiple myeloma. Immunological tests were negative for ANCA, anti-MBG, and ANA. The biopsy showed a significant granular deposit of C3 (+++) in the mesangium and capillary loops. There were positive findings for light and heavy chains in intracellular inclusions of podocytes. Staining was negative for IgG, IgM, IgA, lambda, and fibrinogen. The diagnosis was C3G with focal and segmental sclerosis, collapsing variant, within the context of crystalline podocytopathy caused by kappa light chains (monoclonal gammopathy of renal significance, MGRS). He has low levels of C3 and does not present genetic variations in the complement genes associated with complement-mediated glomerulopaties nor the Δ*CFHR3-CFHR1* polymorphism. He had a negative FHAA ELISA. Reevaluation of this patient with the ICT revealed he is strongly positive for IgG and IgM FH-FHAA complexes.

GN341, a 71-year-old male, was diagnosed with severe primary Raynaud’s syndrome in 2017. By 2019, he exhibited progressive deterioration of kidney function despite having no family history of such conditions. A kidney biopsy, examining 35 glomeruli (three of which were sclerosed), revealed varying degrees of mesangial hypercellularity, focal endocapillary hypercellularity, and a somewhat lobular pattern. The basement membranes were thickened, displaying some isolated double contours. There were no signs of necrosis or capillary thrombi. Immunofluorescence detected abundant granular C3 deposits, likely subendothelial in capillary loops and the mesangium, with negative staining for IgG, IgM, light chains, C1q, and fibrinogen, consistent with a diagnosis of C3G. Between May and December 2019, C3 levels showed activation of the alternative pathway with C3 consumption, but no other abnormalities in other complement proteins were noted. Genetic testing did not reveal pathogenic variants in complement genes associated with glomerular diseases, but the patient was homozygous for Δ*CFHR3-CFHR1*. Throughout 2019, he consistently tested negative for the FHAA ELISA in samples collected on May 28, October 2, and December 17. During this period, the patient was diagnosed with monoclonal gammopathy of uncertain significance, which remained untreated due to the COVID-19 pandemic and due to an unclear relationship to the glomerulopathy. Unfortunately, the patient later experienced a thrombotic microangiopathy event that led to renal failure. He has now received treatment for the gammopathy but continues on peritoneal dialysis and is awaiting a transplant. Reevaluation of this patient with the ICT revealed he is strongly positive for IgG FH-FHAA complexes.

GN198 is a 27y-old female patient with a complex medical history, including a selective IgA deficiency, subclinical hyperthyroidism, and an initial diagnosis of idiopathic thrombocytopenic purpura over 20 years ago, with multiple relapses responding well to immunosuppressive treatments like corticosteroids, rituximab, dexamethasone, and cyclosporine A. Autoimmune tests are positive for ANA, anti-DNA, and ENA (anti-SM and anti-RNP). In 2015, urine tests showed proteinuria of 2.5 grams in a 24-hour urine sample, and a kidney biopsy in 2016 revealed a membranoproliferative pattern with irregular thickening of the capillary wall and subepithelial deposits. Immunofluorescence showed peripheral and clumpy C3 deposits, without IgG, IgM, IgA, and C1q consistent with the diagnosis of C3G. She responded well to low dose steroids, with a serum creatinine of 0.72 mg/dL, an estimated glomerular filtration rate (eGFR) of 121 mL/min, and unquantified proteinuria in 24-hour urine. At that time, she had low serum levels of C3 and C4. Genetic analyses were unremarkable and no copies of Δ*CFHR3-CFHR1*. were observed. She was positive for FHAAs by ELISA but negative for FHAAs by ICT.

Case #18, a 7-year-old male, developed aHUS following a viral illness. At disease onset, he had gross hematuria and proteinuria, as well as thrombocytopenia, which resolved quickly. Although C3 and C4 were normal, elevated C3c and Bb levels indicated C3 convertase hyperactivity. Genetic testing identified compound heterozygosity for two copy number variations, Δ*CFHR3-CFHR1* and Δ*CFHR1-CFHR4*, implying absence of both copies of *CFHR1*. IgG FHAAs were detected by ELISA and ICT. Additionally, ICT strip C identified IgM FHAAs, which ELISA did not detect. The patient has been on Ultomiris (Ravulizumab) since the onset of disease.

Case #34, a 9-year-old male, was hospitalized with septic ankle arthritis and proteinuria with gross hematuria. Over a three-month period, the urine protein-to-creatinine ratio (UPCR) decreased from 1400 mg/g to 270 mg/g and the gross hematuria resolved, although intermittent hematuria was seen in association with four viral illnesses. Persistently low C3, properdin and C5 levels in the face of elevated Bb, and sC5b-9 suggested a complement-mediated renal disease and kidney biopsy confirmed a diagnosis of C3G. Genetic analysis identified a rare *C3* variant (c.4850 +1GG>A) associated with an mRNA splicing defect, resulting in a null allele. In addition, C4 nephritic factors (C4Nefs), C5 nephritic factors (C5Nefs), and FHAAs were detected. The patient tested positive for IgG FHAAs on ICT cassette A, but was negative on ICT cassette B, suggesting binding to the N-terminal region of factor H, as would be predicted in C3G. He also had strong IgM FHAA positivity by ICT, which was not detected by ELISA.

Case #38, a 70-year-old female with end-stage renal disease and a diagnosis of C3G developed nephrotic-range proteinuria and hematuria (urinary protein-to-creatinine ratio 13406 mg/g; blood 3+). Genetic testing identified homozygosity for Δ*CFHR3-CFHR1*. Autoantibody testing indicated strong positivity for IgG FHAAs by both ELISA and ICT; IgM FHAAs were detected exclusively by ICT. The patient was negative for all other known acquired drivers of C3G. Her complement biomarker profile was consistent with dysregulation of the alternative pathway, with persistently low C3 and elevated complement activation products (C3c, C3d, Bb, and sC5b-9). Free factor H levels determined by ELISA were 50% below normal.

### 2.5 Determination of the FH fraction complexed with FHAA

200uL of EDTA-plasma from carriers of FHAA and control individuals were diluted to 4mL with PBS and passed through a 1mL protein G column equilibrated in PBS. 1mL fractions of the flow through were collected (flow through). Concentration of FH in the starting and the flow through samples were determined by ELISA as previously described. The amount of FH complexed with FHAA was calculated by subtracting the FH concentration in the flow through from the original sample. In some cases, FH concentrations were also determined after eluting the IgG bound to the protein G with 100mM Glycine pH 2.5.

### 2.6 Ethics

Plasma or serum from all the patients was available from our biobanks. These samples were originally obtained to perform diagnostic studies and have been kept frozen at -80°C. Approval for storage and usage in novel diagnostic assays was obtained through informed written consent at the time of admission. All studies were approved by our Institute ethics committees.

## 3 Results

**Figure 3:**
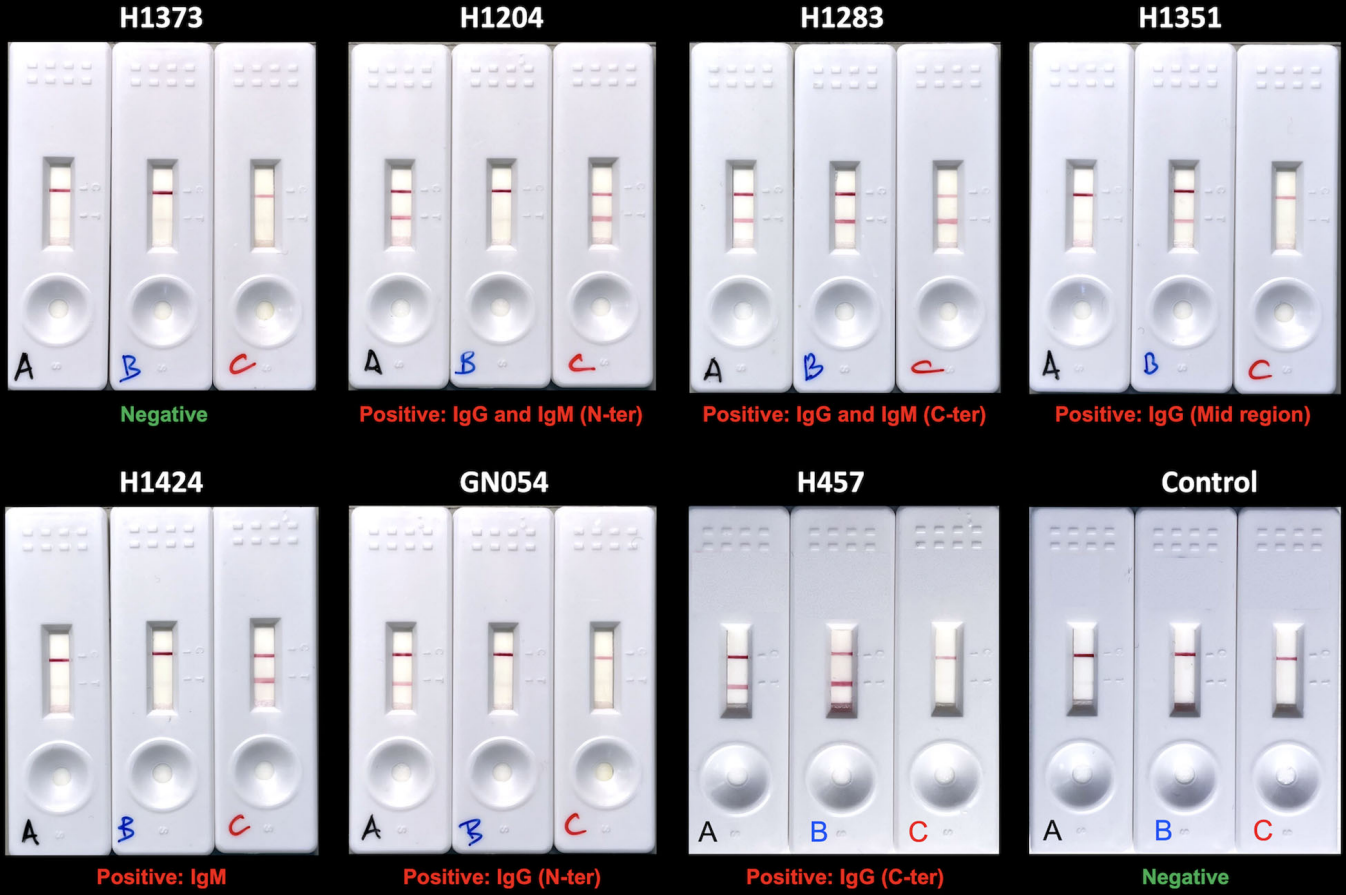
Examples of ICT with negative and positive results. Serum or plasma samples from 6 patients with FH-FHAA complexes involving different types of FHAA and 1 patient negative for FH-FHAA were tested with the three cassettes to illustrate the interpretation of ICT results (show below each trio). A negative control is included in the lower right corner.

**Figure 4:**
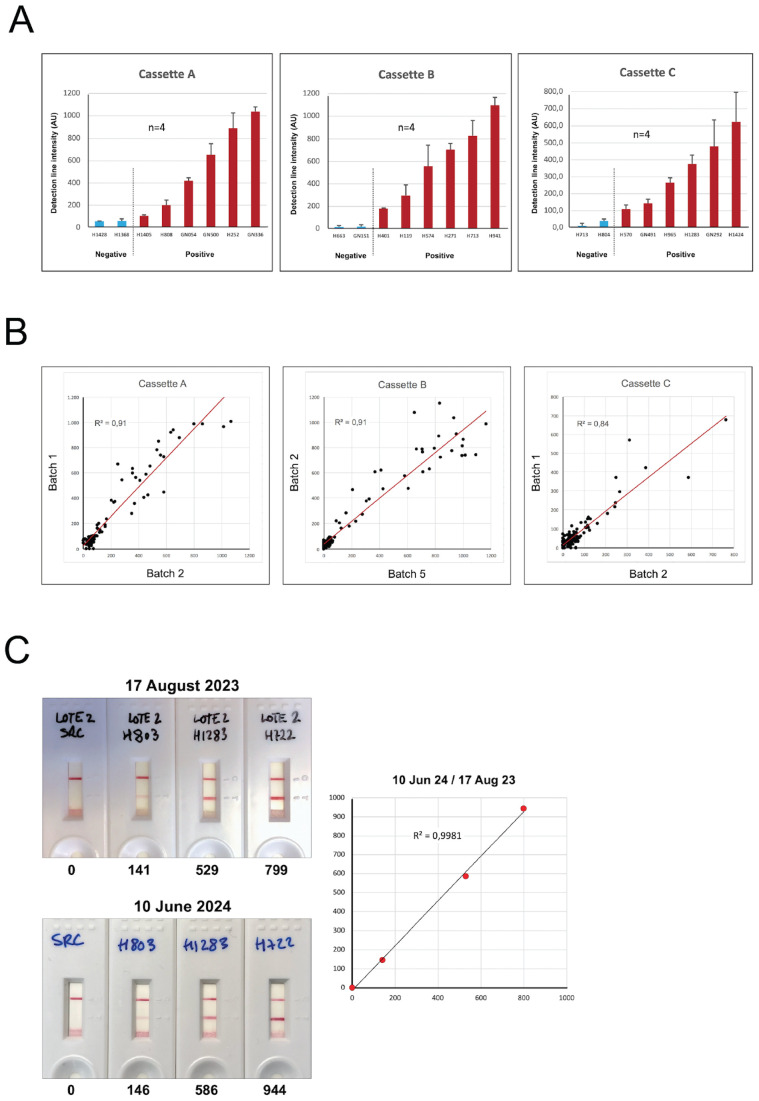
Robustness of the FHAA immunochromatographic test. **(A)** Six positive samples with different levels of FH-FHAA complexes and two negative controls were tested four times with cassettes from the same batch. The results demonstrate excellent reproducibility of the FHAA ICT. **(B)** Similarly, 109 samples from our testing cohort were tested with cassettes A, B and C from two different batches and the results also showed excellent reproducibility. **(C)** Cassettes B from batch 2, generated on June 3, 2023, were used to test the same samples on August 17, 2023, and on June 10, 2024, after being stored in the dark with a desiccant pack at room temperature. They gave essentially identical results, demonstrating excellent stability for at least 1 year. Excel worksheet functions were used for statistical analysis.

To evaluate performance of the FHAA ICT, we generated an aHUS-C3G testing cohort of 155 plasma samples that included 31 positive samples (FHAAs ranging 104-11574 AU), 34 undefined samples (FHAAs ranging 8-97 AU), and 90 negative samples (AU=0); 58 samples were homozygous for Δ*CFHR3-CFHR1*. None of the 155 samples had been tested for FHAA of the IgM type and epitope mapping had not been done. All positive and “undefined” samples and 44 negative samples in this testing cohort were analyzed with the three cassettes (A, B and C) from two different batches prepared 6 months apart (See examples of positive and negative results in **Figure 3**). Selected samples were also tested repeatedly using various cassettes from the same batch. Cassettes A, B and C show excellent reproducibility within and between batches and were stable for at least 12 months (**Figure 4**).

A comparison between ELISA and the ICT results illustrates that of the 31 FHAA(IgG) ELISA-positive samples, 28 were positive for FH-FHAA complexes by ICT (**Table 1**). Two samples (HUS1288 and GN241) were weakly positive and one (GN198) was clearly negative. The discrepancy between the ELISA and the ICT results with sample GN198 was consistently observed in multiple repetitions of these assays. ICT identified 4 IgG positive samples that were also positive for FH-FHAA(IgM) complexes; two of these samples (HUS1204, HUS1283) were strong positives and two (HUS941, HUS119) were weak positives. Epitope mapping by ICT showed that in three positive cases (HUS1204, GN351 and GN054) the FHAA recognized the N-terminal region of FH and in one case (HUS1351), the mid-region of FH (**Table 1**).

**Table 1:** ICT results in samples that tested positive (AU>100) in the FHAA ELISA.

				IMMUNOCHROMATOGRAPHIC DATA (AU Detection Line)*											
				Batch 1			Batch 2								
DISEASE	*Δ_CFHR3-CFHR1_*	SAMPLE	ELISA (AU)	A	B	C	A	B	C (AU)	INTERPRETATION IMMUNOCHROMATOGRAPHIC DATA					
aHUS	HOM	HUS 533	112	113	327	41	198	390	57	POS	IgG				C-TER
aHUS	HET	HUS 1204	119	250	40	312	666	90	569	POS	IgG	IgM	N-TER		
aHUS	HOM	HUS 013	131	221	579	38	362	573	64	POS	IgG				C-TER
aHUS	HOM	HUS 1288	158	87	114	55	102	202	36	Weak POS	IgG				C-TER
aHUS	HOM	HUS 119	164	100	307	110	160	373	104	POS	IgG	IgM weak			C-TER
aHUS	HOM	HUS 1351	246	79	207	35	72	464	6	POS	IgG			Mid-region	
aHUS	HOM	HUS 271	278	279	757	16	541	630	33	POS	IgG				C-TER
aHUS	NT	HUS 646	291	230	606	30	370	473	35	POS	IgG				C-TER
aHUS	HOM	HUS 246	372	454	707	64	587	786	48	POS	IgG				C-TER
aHUS	HOM	HUS 471	508	482	996	0	650	812	50	POS	IgG				C-TER
aHUS	HOM	HUS 653	793	357	920	20	632	773	23	POS	IgG				C-TER
aHUS	HOM	HUS 154	1013	203	712	21	380	605	39	POS	IgG				C-TER
aHUS	HOM	HUS 472	1083	409	708	2	537	764	44	POS	IgG				C-TER
aHUS	HOM	HUS 713	1120	532	838	0	781	721	8	POS	IgG				C-TER
aHUS	HOM	HUS 1318	1561	560	934	25	739	1033	20	POS	IgG				C-TER
aHUS	HOM	HUS 108	1648	356	952	39	597	904	50	POS	IgG				C-TER
aHUS	HOM	HUS 252	2679	800	832	0	986	1149	35	POS	IgG				C-TER
aHUS	NO	HUS 1283	2715	380	412	387	560	618	423	POS	IgG	IgM			C-TER
aHUS	HET	HUS 151	3406	633	1091	60	920	741	0	POS	IgG				C-TER
aHUS	HOM	HUS 941	3528	544	1014	115	848	738	152	POS	IgG	IgM weak			C-TER
aHUS	HOM	HUS 457	4809	469	666	41	420	785	19	POS	IgG				C-TER
aHUS	HOM	HUS 722	7898	1014	1164	29	966	987	64	POS	IgG				C-TER
aHUS	HOM	HUS 574	8895	582	368	35	444	607	22	POS	IgG				C-TER
aHUS	HOM	HUS 177	11574	694	993	52	877	736	51	POS	IgG				C-TER
C3G	NO	GN 336	117	1067	792	31	1006	794	72	POS	IgG				C-TER
C3G	HOM	IND 081	122	120	276	5	145	272	21	POS	IgG				C-TER
C3G	NO	GN 147	172	649	1001	41	938	864	115	POS	IgG				C-TER
C3G	NO	GN 351	269	351	28	6	276	44	42	POS	IgG		N-TER		
C3G	NO	GN 198	355	22	47	7	0	30	0	NEG					
C3G	NO	GN 241	373	105	90	5	156	220	27	Weak POS	IgG				C-TER
C3G	HET	GN 054	3081	438	28	52	401	37	61	POS	IgG		N-TER		

*) AU, arbitrary units. Positive values in the detection band are depicted in red; values above 100AU are considered positive using the Colloidal Gold Rapid Test Strip Reader CHL-TSR100 from Guangzhou Iclear Healthcare Limited. NO, no carrier of ***Δ***_*CFHR3-CFHR1*_. NT, not tested.

ICT was most informative in the 34 samples classified as “undefined” by ELISA. Two samples (HUS965 and GN292) were strongly positive for FH-FHAA(IgM) complexes and 3 samples (HUS663, HUS164 and GN341) were strongly positive for FH-FHAA(IgG) complexes, with HUS663 containing FHAAs directed to the N-terminal region of FH. 6 samples were weakly positive for FH-FHAA(IgG) complexes, with FHAAs directed in 2 samples to the N-terminal region and in 2 samples to the mid-region of FH (**Table 2**).

**Table 2:** ICT results in samples that tested “undefined” (0<AU<100) in the FHAA ELISA.

				IMMUNOCHROMATOGRAPHIC DATA (AU Detection Line)*											
				Batch 1			Batch 2								
DISEASE	*Δ_CFHR3-CFHR1_*	SAMPLE	ELISA (AU)	A	B	C	A	B	C	INTERPRETATION IMMUNOCHROMATOGRAPHIC DATA					
aHUS	HOM	HUS 749	8	137	231	67	131	216	48	Weak POS	IgG				C-TER
aHUS	NO	HUS 552	9	33	21	31	64	26	44	NEG					
aHUS	HET	HUS 110	14	45	51	49	87	86	41	NEG					
aHUS	NO	HUS 803	16	99	159	44	185	280	62	Weak POS	IgG				C-TER
aHUS	NO	HUS 613	18	66	31	32	67	76	42	NEG					
aHUS	HET	HUS 848	19	15	0	58	34	1	30	NEG					
aHUS	NO	HUS 663	24	370	22	72	355	51	32	POS	IgG		N-TER		
aHUS	NO	HUS 1095	25	50	36	51	84	45	69	NEG					
aHUS	NO	HUS 1325	27	54	44	39	94	31	42	NEG					
aHUS	NO	HUS 265	27	45	37	43	57	19	17	NEG					
aHUS	NO	HUS 1183	29	44	0	31	0	25	83	NEG					
aHUS	HOM	HUS 401	29	71	183	47	88	176	18	Weak POS	IgG			Mid-region	
aHUS	NO	HUS 804	29	43	0	28	35	0	27	NEG					
aHUS	NO	HUS 349	31	63	17	86	64	13	95	NEG					
aHUS	HET	HUS 1328	33	85	50	66	94	83	58	NEG					
aHUS	NO	HUS 855	38	61	11	52	98	39	40	NEG					
aHUS	HET	HUS 644	43	34	21	45	57	36	35	NEG					
aHUS	NO	HUS 570	57	60	28	106	118	94	88	NEG					
aHUS	NO	HUS 965	48	72	0	266	100	62	294	POS		IgM			
aHUS	HET	HUS 797	63	160	43	54	171	91	62	Weak POS	IgG		N-TER		
aHUS	NO	HUS 808	64	169	79	30	233	91	41	Weak POS	IgG		N-TER		
aHUS	NO	HUS 999	67	65	0	67	80	37	97	NEG					
aHUS	NO	HUS 1405	70	86	34	72	99	38	50	NEG					
aHUS	HET	HUS 829	80	33	35	125	38	50	90	NEG					
aHUS	HOM	HUS 164	85	159	426	44	184	471	33	POS	IgG				C-TER
aHUS	NO	HUS 525	97	33	46	51	43	47	50	NEG					
C3G	HOM	GN 341	9	860	826	39	987	889	29	POS	IgG				C-TER
C3G	HET	GN 292	38	40	41	588	90	23	371	POS		IgM			
C3G	NT	IND 065	46	67	34	9	81	42	15	NEG					
C3G	NO	GN 151	52	74	7	0	128	36	16	NEG					
C3G	HOM	GN 263	52	70	124	41	72	160	42	Weak POS	IgG			Mid-region	
C3G	NO	GN 368	52	57	37	66	86	50	46	NEG					
C3G	NO	GN 191	53	74	35	47	0	23	35	NEG					
C3G	NO	GN 364	67	56	47	44	49	53	40	NEG					

*) AU, arbitrary units. Positive values in the detection band are depicted in red; values above 100AU are considered positive using the Colloidal Gold Rapid Test Strip Reader CHL-TSR100 from Guangzhou Iclear Healthcare Limited. NO, no carrier of *Δ_CFHR3-CFHR1_*. NT, not tested.

Of the 90 ELISA FHAA negative samples, all except 1 (GN500) were also negative for FH-FHAA complexes of the IgG type by ICT. Notably, sample GN500 was also positive for FH-FHAA complexes of the IgM type, as were 9 additional patients, who were positive for only IgM FH-FHAA complexes, 6 of them weakly (**Supplementary Table 1**).

As described, ICT provided information regarding the presence of IgM-FHAAs, which we cannot detect by our FHAA ELISA. In total, 16 patients in our testing cohort carried IgM FHAAs: 5 patients were co-positive for IgG FHAAs and 11 were positive for only IgM FHAAs (5 aHUS and 6 C3G). Of these 16 cases, 6 had prominent intensities at the detection line indicating significant levels of IgM FH-FHAA complexes. Interestingly, 4 of 5 weakly positive cases did not carry the deletion Δ*CFHR3-CFHR1*, while amongst the 6 clear positives samples, 2 are homozygous and 2 are heterozygous for the deletion (**Tables 1**, **2**; **Supplementary Table 1**). These data suggest that amongst aHUS and C3G patients, a significant percentage of patients present with IgM FHAAs antibodies that drive disease, which fits well with recent published data reporting the presence of IgM FHAA in these pathologies (14, 15).

We also tested samples from 40 healthy controls and all of them were negative for FH-FHAA complexes in the ICT. These samples were useful to set up the positive cut off value at >100AU when reading the ICT with the Colloidal Gold Rapid Test Strip Reader.

### 3.1 Validation of ICT epitope mapping and IgM data

Since none of the samples included in our testing cohort had been tested for IgM FHAAs by ELISA and we did not perform epitope mapping in those found IgG FHAA positive by ELISA, we used an additional group of samples selected from those analyzed in the Molecular Otolaryngology and Renal Research Laboratories at the University of Iowa to confirm that our interpretation of the results of the ICT were correct. Thirty-one IgG FHAA-positive samples by ELISA epitope mapping data were included in these validation analyses. IgG FHAA ICT results (cassettes A and B) were consistent with IgG FHAA ELISA results, with all 31 patients testing positive on strips A and/or B. Notably, in all samples, the ICT results align with epitope mapping data determined by ELISA (**Table 3**). Thus, samples with C-terminal binding FHAA which were verified by ELISA epitope mapping exhibited an ICT reading pattern with positive results in the A and B cassettes and similar intensities in the detection bands of both cassettes. In contrast, in samples with N-terminal binding FHAAs, cassette B was negative or the intensity of the detection band significantly lower than that of cassette A (A>>B). Samples with a reading pattern of positive results in cassette B and negative or very low positivity in cassette A (A<<B) are indicative of FHAA binding the mid region of FH. In aggregate, data obtained with the validation samples indicate that epitope information provided by ICT testing is reliable.

**Table 3:** Validation of ICT epitope mapping data.

			ELISA	ELISA	ICT DATA (Detection Line)*								
DISEASE	*Δ_CFHR3-CFHR1_*	SAMPLE	IgG (AU)	EPITOPE MAPPING	A (AU)	B (AU)	C (AU)	INTERPRETATION ICT DATA					
aHUS	HOM	1	3136	C-ter FH 18-20	19	66		Pos	IgG			Mid region?	C-TER
aHUS	HOM	2	1259	C-ter FH 18-20	32	102		Pos	IgG				C-TER
aHUS	HET	3	33190	N-ter FH 1-5; C-ter FH 18-20	78	55		Pos	IgG				C-TER
aHUS	HOM	6	5961	C-ter FH 18-20	101	129		Pos	IgG				C-TER
aHUS	NO	7	2250	N-ter FH 1-5	45	16		Pos	IgG		N-TER?		
aHUS	HOM	8	1149	C-ter FH 18-20	47	101		Pos	IgG				C-TER
aHUS	HOM	9	1917	C-ter FH 18-20	97	135		Pos	IgG				C-TER
aHUS	HOM	10	1522	C-ter FH 18-20	63	116		Pos	IgG				C-TER
aHUS	HET	11	25130	C-ter FH 18-20	79	121		Pos	IgG				C-TER
aHUS	HOM	14	2062	C-ter FH 18-20	85	121		Pos	IgG				C-TER
aHUS	NT	17	13930	N-ter FH 1-5	48	4		Pos	IgG		N-TER		
aHUS	HET	18	10620	C-ter FH 18-20	94	134		Pos	IgG				C-TER
aHUS	HOM	19	10900	C-ter FH 18-20	88	117		Pos	IgG				C-TER
aHUS	NT	20	35490	C-ter FH 18-20; FH 8-15	70	104		Pos	IgG				C-TER
aHUS	HETdelCFHR1-4	21	1103	C-ter FH 18-20	27	61		Pos	IgG				C-TER
aHUS	HOM	22	2182	C-ter FH 18-20	33	70		Pos	IgG				C-TER
aHUS	HET	23	6361	C-ter FH 18-20	90	126		Pos	IgG				C-TER
aHUS	HOM	24	50070	C-ter FH 18-20	101	131		Pos	IgG				C-TER
aHUS	HOM	25	3087	C-ter FH 18-20	49	97		Pos	IgG				C-TER
aHUS	NO	26	5019	C-ter FH 18-20	53	75		Pos	IgG				C-TER
aHUS	HOM	27	13550	all-SCRs	37	79		Pos	IgG				C-TER
C3G	HOM	29	5620	N-ter FH 1-5; C-ter FH 18-20	80	71		Pos	IgG				C-TER
C3G	NT	34	1106	N-ter FH 1-5; FH 8-15	23	9		Pos	IgG		N-TER		
C3G	HOM	38	12120	C-ter FH 18-20	100	104		Pos	IgG				C-TER
C3G	HETdelCFHR1-4	40	6015	N-ter FH 1-5; Very weak C-ter 18-20	73	30		Pos	IgG		N-TER?		C-TER
C3G	HET	41	6015	N-ter FH 1-5; FH 6-8, C-ter FH 18-20	51	46		Pos	IgG				C-TER
C3G	NO	43	1342	N-ter FH 6-8	108	99		Pos	IgG				C-TER
C3G	HOM	45	3087	C-ter FH 18-20	31	104		Pos	IgG				C-TER
C3G	HET	46	5041	N-ter FH 1-5	56	12		Pos	IgG		N-TER		
GMRS	NO	48	9089	N-ter FH 1-5	44	3		Pos	IgG		N-TER		
GMRS	HOM	51	46180	C-ter FH 18-20	118	128		Pos	IgG				C-TER

*) AU, arbitrary units. Positive values in the detection band are depicted in red; values above 12AU are considered positive using the ESEQuant Flex equipment from DIALUNOX GmbH. MGRS, Monoclonal gammopathy of renal significance. NO, no carrier of *Δ_CFHR3-CFHR1_*. NT, not tested.

Five IgM FHAA-positive samples from the Molecular Otolaryngology and Renal Research Laboratories at the University of Iowa were used to validate the ICT IgM data. We found that all samples positive for IgM FHAA by ELISA were also detected by the ICT strip C, demonstrating that the ICT is suitable for identifying FHAA immune complexes in patients with IgM FHAA (**Table 4**).

**Table 4:** Validation of ICT IgM data.

Disease	*Δ_DCFHR3-CFHR1_*	Sample	ELISA		ICT data (Detection Line)*			Interpretation ICT data		
			IgG (AU)	IgM (OD)	A (AU)	B (AU)	C (AU)			
C3G	HET	41	6015	1,69	51	46	24	Pos	IgG	IgM
C3G	HET	46	5041	1,16	56	12	50	Pos	IgG	IgM
GMRS	NO	50	<50	3,5	27	4	80	Pos	IgG	IgM
Unknown	NT	58	<50	0,98	8	6	25	Pos	–	IgM
Unknown	NT	59	61	1,67	7	5	22	Pos	–	IgM

*) Positive values in the detection band are depicted in red; values above 12AU are considered positive using the ESEQuant Flex equipment from DIALUNOX GmbH. MGRS, Monoclonal gammopathy of renal significance. NO, no carrier of ***Δ***_CFHR3-CFHR1_. NT, not tested.

Interestingly and similarly to the detection of IgG FHAA with the ICT in samples that tested negative by ELISA in the testing cohort, we also identified some samples that were clearly positive for IgM FHAA in the ICT but negative by ELISA.

### 3.2 ICT results correlate better with levels of FH-FHAA complexes than do free FHAA titers

**Figure 5:**
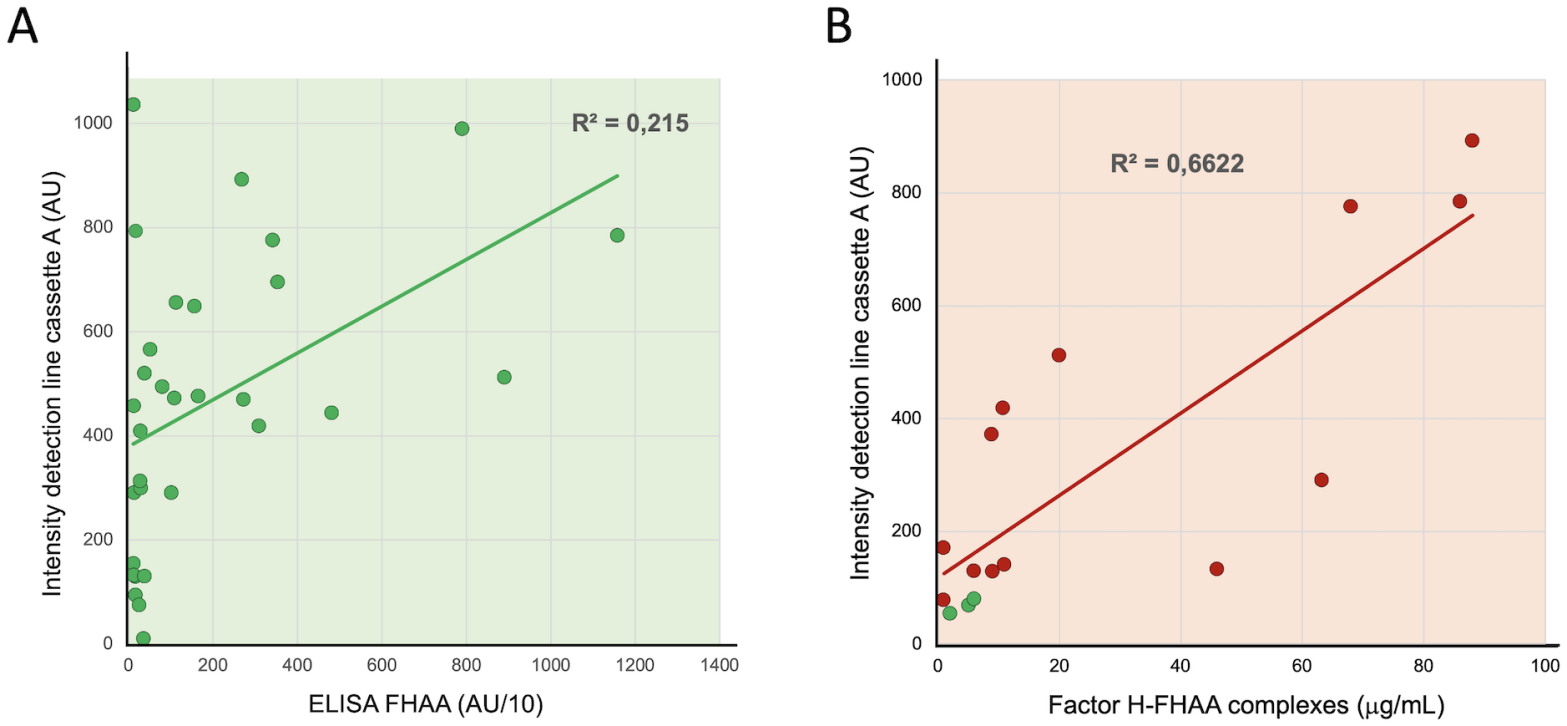
Correlation between the intensity of the detection line with free FHAA titers and FH-FHAA complexes. **(A)** Autoantibody titers in all samples testing positive by ELISA (AU>100) (**Table 1**) were plotted vs the average of the two measurements of the intensity of the detection band in cassette A. **(B)** For selected samples, positives by ICT (red dots), the plasma concentration of FH before and after passing through a protein G column was determined to estimate the amount of FH retained in the IgG fraction due to formation of FH FHAA complexes. These FH concentrations were plotted vs the intensity of the detection band in cassette A. Three negative controls were also included in these experiments (green dots). See also **Table 5**.

The analyses of our testing cohort illustrate that there is no or only a low correlation between the titers of the free FHAA detected by the ELISA and the intensity of the detection line by the ICT (**Figure 5A**). A likely explanation for this observation may be differences in the affinity of the FHAAs for FH (16). To determine how the intensity of the detection band correlates with the levels of FH-FHAA complexes in the sample, as well as to provide an understanding of the sensitivity of the ICT, we determined the amount of FH-FHAA(IgG) complexes in selected samples (**Table 5**) as described in Materials and Methods. These analyses showed a good correlation between the color intensity of the detection line and the amounts of FH-FHAA complexes (**Figure 5B**). The analysis also shows that ICT offers excellent sensitivity to the detection of FH-FHAA complexes and, in practical terms, demonstrates that ICT provides strong positive results when more than 10ug/mL of FH is complexed with FHAA. For a sample with plasma FH levels within the normal range (90-250µg/mL) this amount corresponds to a percentage of FH complexed with FHAA between 4% and 10%.

**Table 5:** FH concentration, ELISA and ICT data in samples used in the protein G experiments.

	Plasma (µg/mL)	Flow through (µg/mL)	Bound (P-F) (µg/mL)	ICT (Cassette A)*	ELISA (FHAA)
BMT	171	166	5	69	0
HMM	103	97	6	80	0
SRC	172	170	2	54	0
GN054	159	148	11	420	3081
H574	150	130	20	513	8895
H151	131	63	68	777	3406
H154	128	65	63	292	1013
H252	146	58	88	893	2679
H177	108	22	86	786	11574
H803	79	68	11	142	16
GN241	97	91	6	131	373
H401	187	185	1	80	29
H164	111	119	1	172	85
H663	124	115	9	373	24
H749	161	115	46	134	8
H119	99	90	9	130	164

*) Values are the average of the two cassette A measurements depicted for these cases in **Tables 1** and **2**.

### 3.3 Discrepancies between FHAA ELISA and ICT

The analysis of 155 aHUS-C3G plasma samples has revealed that with respect to IgG FHAAs, ICT identified 4 clearly FH-FHAA(IgG) positive samples (HUS663, HUS164, GN341 and GN500) and 6 weakly positive samples that were missed by ELISA. While ELISA identified 1 positive sample (GN198) that was negative by ICT and 2 positive samples (HUS1288 and GN241) that were only weakly positive by ICT. GN341, GN500 and GN198 samples were available for further analysis and their clinical data are summarized briefly in Material and Methods.

**Figure 6:**
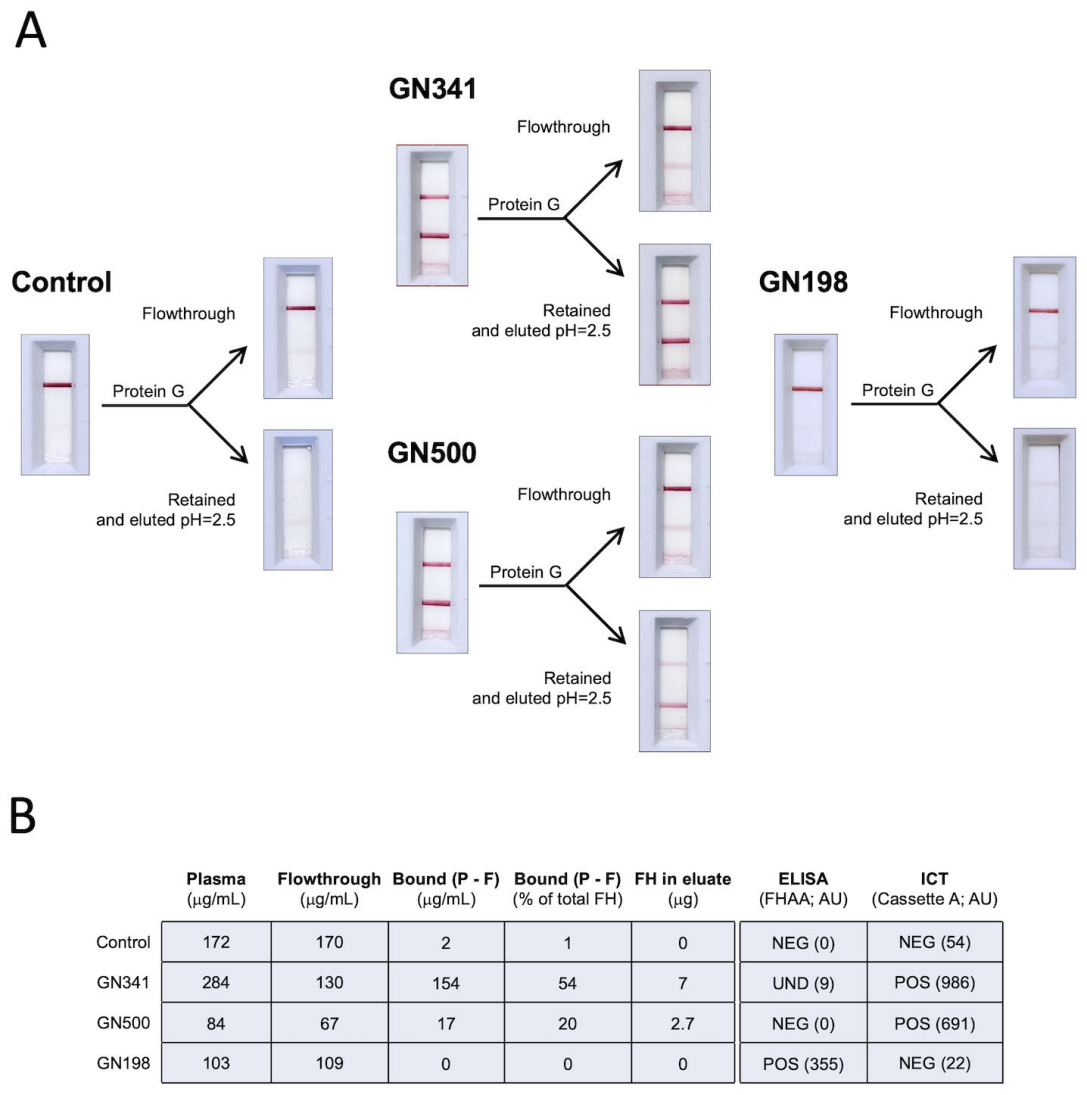
Characterization of IgG FH-FHAA complexes in samples with conflicting ELISA and ICT results. **(A)** Samples from GN341, GN500, GN198 and from a negative control were passed through a protein G column and the FH concentration was determined in the flow through and the eluate for each sample. The amount of FH retained in the column was determined by subtracting the concentration of FH at the plateau of the elution peak as a more reliable measurement since a variable amount of FH in each sample is lost during the column washes. Cassette B was used to test for the presence of FH-FHAA complexes in the original, the flow through, and the eluted samples. FH-FHAA complexes present in GN341 and GN500 were clearly reduced in the flow through and appear in the eluate, while they were absent in all fractions from GN198 and the control samples. **(B)** FH concentrations in all samples are shown, together with the ELISA and ICT data. Notice that no FH is detected in the eluate from the GN198 and the control samples. Normal FH range: 90-285µg/ml. NEG, Negative; POS, positive; UND, Undefined.

Protein G purification of the IgG fraction from EDTA-plasma of GN341 and GN500 showed that a significant amount of FH was removed with the IgG bound to the protein G (54% and 20%, respectively), illustrating the presence of significant amounts of FH-FHAA (IgG) complexes in these two samples and suggesting that failure to detect FHAAs by ELISA is due to the absence of free FHAA in these samples (**Figure 6**). This finding may imply that patients carrying low titers of FHAA with high affinity for FH will escape detection of clinically relevant FHAA by traditional ELISA methods. Notably, in these two patients a monoclonal gammopathy was detected that may explain the peculiarities of their FHAA. In contrast, purification of the IgG fraction from the EDTA-plasma of GN198 using a protein G column failed to demonstrate the presence of FH-FHAA complexes, as no FH could be detected in the IgG fraction retained by the protein G (**Figure 6**). Because plasma FH levels in this patient were normal, our conclusion is that GN198 is likely a false positive of our free FHAA ELISA and may be related to the complex autoimmune condition in this patient.

**Figure 7:**
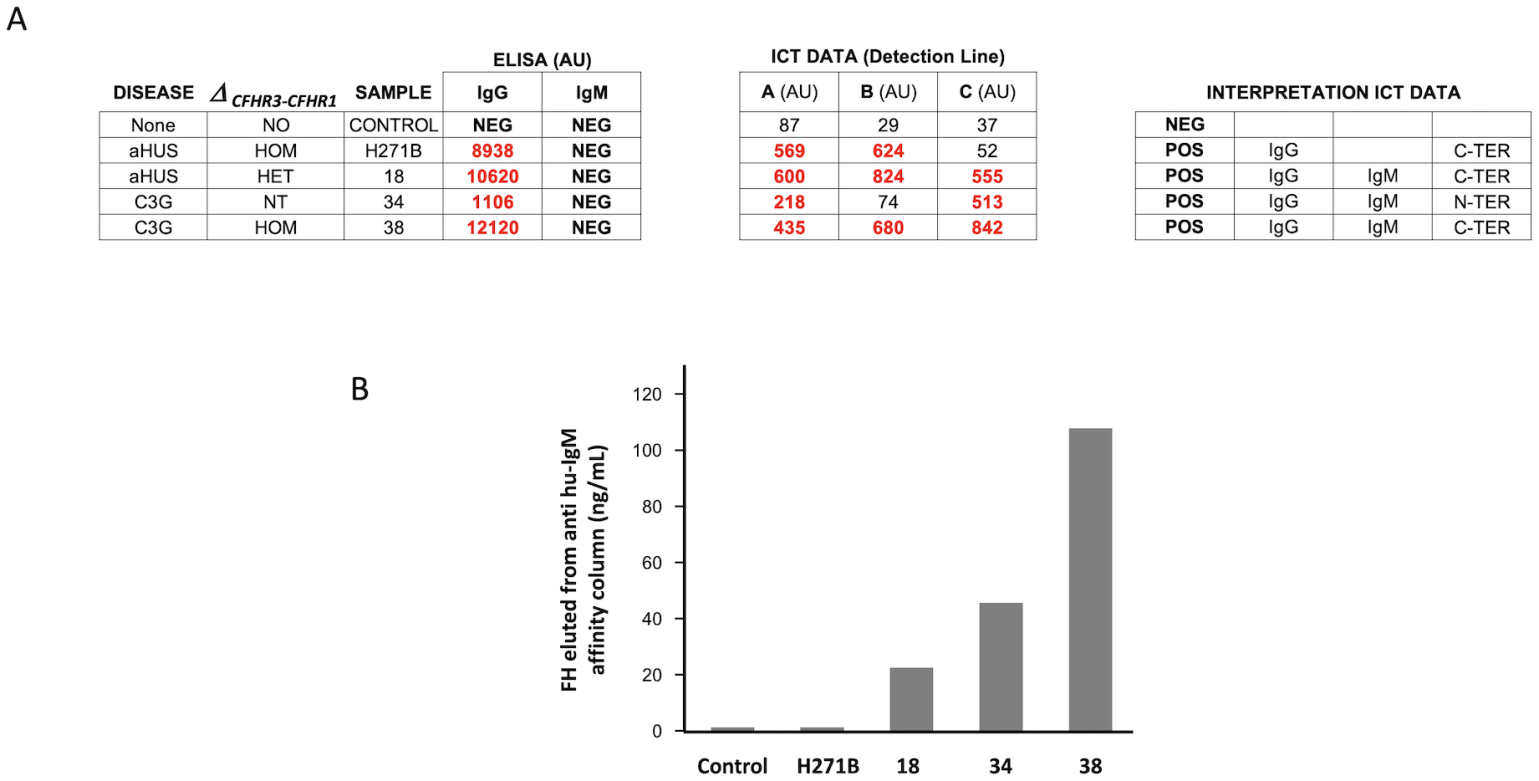
Characterization of IgM FH-FHAA complexes in samples with conflicting ELISA and ICT results. **(A)** Results of the analisis by the ICT of plasma samples from cases #18, #34 and #38, from a healthy control (IgG/IgM FHAA negative plasma) and from a strong positive plasma for IgG FHAA that was negative for IgM FHAA (H271B). **(B)** Plasma from these five samples was passed through an affinity column coated with a goat polyclonal anti-human IgM (Fc fragment) that specifically captures human IgM antibodies and the eluate for each sample tested for the presence of FH by ELISA. The results of these experiments show that while no FH was retained from the healthy control plasma nor the strong positive plasma for IgG FHAA, significant amounts of FH were eluted from the column in the case of the case of the three samples that tested positive for IgM FH-FHAA complexes in the ICT.

Similar experiments were performed in three cases (#18, #34 and #38) in which the ICT identified the presence of significant titers of IgM FH-FHAA complexes, but were negative for IgM FHAA in the ELISA. In these experiments we prepared an affinity column using a goat polyclonal anti-human IgM (Fc fragment) to specifically capture human IgM antibodies and tested whether FH was also retained by the column when EDTA plasma from cases #18, #34 and #38 was passed through. For controls we used an IgG/IgM FHAA negative plasma from a healthy control and a strong positive plasma for IgG FHAA that was negative for IgM FHAA (H2718). The results of these experiments show that while no FH was retained from the healthy control plasma nor the strong positive plasma for IgG FHAA, significant amounts of FH were eluted from the column in the case of the case of the three samples that tested positive for IgM FH-FHAA complexes in the ICT (**Figure 7**).

In whole these data suggest that the ICT cassettes are more sensitive than ELISA methods to detect IgG and IgM FHAA and illustrate that in some patients with FH-FHAA complexes, free FHAA may not be detected by ELISA.

### 3.4 Monitorization of FH-FHAA complexes in a patient under immunosuppression treatment

**Figure 8:**
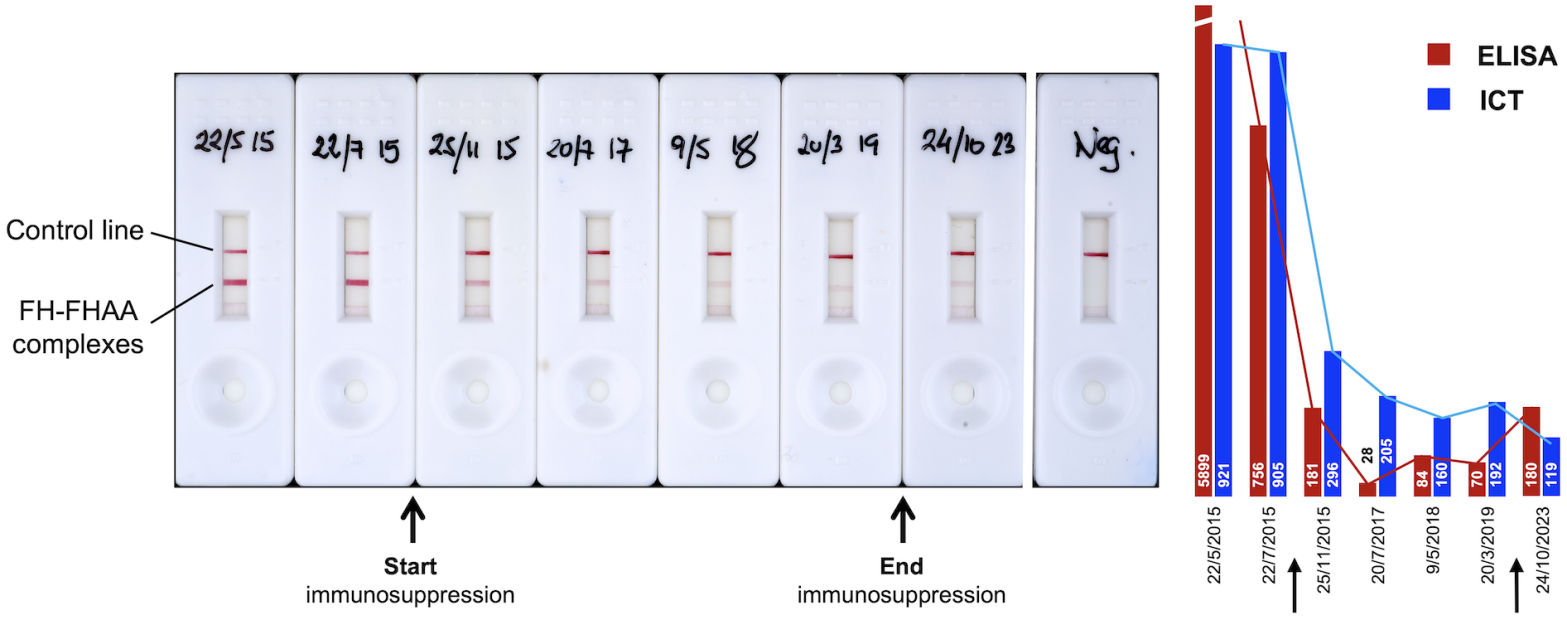
Use of ICT to monitor FH-FHAA complexes in patients under immunosuppressive treatment. Serial samples from a patient originally diagnosed with high free FHAA titers, as determined by ELISA, were obtained at different dates (hand written in the cassettes), before, during, and after immunosuppression. For each sample, an ELISA and ICT were performed to determine titers of free FHAA and levels of FH-FHAA complexes.

Because an important application of the ICT would be to follow FHAA levels in patients under immunosuppressive treatment, we tested serial samples in a FHAA-positive patient treated with immunosuppression and documented the capacity of ICT to detect variations in the levels of FH-FHAA complexes. This observation clearly illustrates that ICT is not inferior to ELISA methods in monitoring these patients - the decrease in the levels of FH-FHAA complexes as detected by ICT mirrored that of the free FHAA titers as measured by ELISA. Both assays show that these levels remain low years after the immunosuppression was discontinued (**Figure 8**).

## 4 Discussion

FHAAs are of specific importance to aHUS and C3G as their detection is critical in the diagnosis, management, and targeted treatment of these conditions. Their presence significantly affects both immediate treatment strategies and long-term outcomes. Monitoring FHAAs levels aids in evaluating treatment effectiveness, informing duration of therapy, and prognosticating outcome.

Current FHAA diagnostic methods are ELISA-based and, typically, they are limited to detecting free FHAAs using a densely FH-coated surface to which free autoantibodies bind. While these methods provide excellent sensitivity, they cannot inform how much circulating FH is blocked by FHAAs. Because FH-FHAA complexes reflect the actual involvement of FHAA in the pathogenic process, they may provide a more robust correlation with clinical outcomes, leading to more precise diagnostics and timely interventions. ICT offers an excellent methodology for the real-time detection of circulating FH-FHAA complexes. As samples flow through the test strip, ICT leverages the movement of fluids to facilitate the immediate interaction between capture and detection antibodies with pre-existing FH-FHAA complexes in the sample. The quick visualization of results, typically within minutes, makes ICT an invaluable tool in scenarios requiring prompt decision-making, such as in clinical diagnostics. The speed and simplicity of these tests, combined with their ability to operate without sophisticated equipment, make them especially useful in point-of-care settings and in resource-limited environments.

The ICT described herein is the first visual method for the rapid detection and quantification of IgG and IgM FH-FHAA complexes in human EDTA-plasma or serum. Results from comprehensive evaluation of the performance of ICT as compared to the ELISA method developed in our laboratory show that ICT is not only very robust both in terms of stability and reproducibility of results (**Figure 4**), but also that it offers similar sensitivity to detect FHAAs as compared to the ELISA. Notably, the ICT shows increased specificity compared to the ELISA, detecting false-negative and false-positive ELISA cases. In fact, the study highlights a notable discrepancy between the FHAA ELISA and the FHAA ICT in some instances.

Among the 31 samples positive for FHAA(IgG) by ELISA, the ICT confirmed the presence of FH-FHAA complexes in 30 (2 weakly positive in the ICT), indicating a good alignment in most cases. However, critical exceptions like sample GN198, which tested positive in ELISA but negative for FH-FHAA complexes in all ICT repetitions, or GN341 and GN500, identified by the ICT as FHAA positive samples that the ELISA missed, suggest a potentially higher sensitivity and specificity of the ICT. The discrepancies observed between the two testing methods, especially in samples like GN198, GN341, and GN500, suggest that the ability of ICT to detect complexes rather than just free antibodies may offer a more accurate approach to characterize the presence of FHAAs and to explain the pathological state in patients.

A particular diagnostic improvement in our aHUS-C3G cohort afforded by ICT was the detection of IgM-type FHAAs in 16 samples, which our FHAA ELISA could not detect as it does not target IgM-type FHAAs. These samples included 5 co-positive for both IgM and IgG FHAAs and, most important, 11 samples solely positive for IgM FHAAs. Six of these latter 11 samples had significant amounts of circulating FH-FHAA complexes (**Tables 1**, **2**; **Supplementary Table 1**). As for the detection of IgG FHAAs, discrepancies in the detection of IgM-type FHAAs were also observed between the two testing methods, like cases #18, #34 and #38, again suggest that the ability of ICT to detect FH-FHAA complexes rather than just free antibodies may offer a more accurate approach to characterize the presence of IgM FHAAs.

Notably, ICT also provided insights into the epitope specificity of the identified FHAAs, particularly detecting antibodies targeting the N-terminal and mid-region of Factor H. This kind of detailed epitope mapping, which is not provided by standard FHAA ELISA methods, could enhance the understanding of disease mechanisms and potentially guide more targeted therapies. Finally, the correlation between the intensity of the ICT detection line and the quantity of FH-FHAA complexes (as shown in **Figure 5B**) supports the practical sensitivity and reliability of the ICT in detecting FH-FHAA complexes quantitatively. It also highlights its potential utility in longitudinal disease management by aiding in monitoring patients under immunosuppressive therapy (**Figure 8**).

Our test allows the detection of FH-FHAA complexes of the IgG and IgM types, as well as epitope mapping, which represent an extension beyond the current routine practice of detecting only free FHAA. We believe this approach would provide a more accurate estimation of the contribution of acquired risk factors (anti-FH autoantibodies) in identifying the etiological factors underlying complement-mediated diseases such as aHUS and C3G and would be particularly valuable in the context of monoclonal gammopathy.

One limitation of our study is that we may not have included a sufficient number of controls to reliably estimate the false positive rate. In our FHAA ELISA-tested cohort, we identified several positives using the ICT for both IgG and IgM that were negative in the ELISA. In the cases we were able to analyze further, we demonstrated that the ICT results were true positives, suggesting that this method is both cleaner and more sensitive than ELISA approaches for measuring IgG and IgM FHAA. However, our data on ICT-positive results in the general control population is limited. Thus far, we have found none, but further studies are needed to clarify their existence and, if present, their significance. Notably, these analyses would be particularly critical in control populations of ethnic groups where the incidence of FHAA is especially high.

Our ICT offers both a quicker and more economical alternative to the ELISA test. For ELISA to be cost-effective, it requires the simultaneous testing of multiple samples, which inevitably leads to significant delays in delivering results. Additionally, FHAA testing is not universally available and is often limited to a small number of reference laboratories. Shipping samples to these laboratories, when possible, introduces further delays and increases costs.

We acknowledge that the full value of the ICT will only be realized when it is tested across multiple laboratories. To that end, as indicated in the Data availability statement of this manuscript, we offer the ICT to anyone interested in evaluating it and providing feedback.

In conclusion, the ICT we have developed and described offers the first visual method to easily and rapidly detect and quantify IgG and IgM FHAAs by measuring the levels of FH-FHAA complexes in human EDTA-plasma or serum. This ICT offers improved specificity compared to traditional ELISA methods as demonstrated by cases where ICT identifies the presence of FH-FHAA complexes in samples negative for FHAAs by ELISA. Importantly, ICT indirectly informs on the amount of FH that is complexed with FHAA, thus assessing the significance of the FHAA to the pathology. This novel assay will provide a simpler, cost-effective, faster, and more clinically relevant alternative for diagnosing FHAA in at-risk populations. The ability of the ICT to detect and quantify various FH-FHAA complexes and its utility in monitoring treatment efficacy make it a compelling option for broad adoption in clinical settings, ensuring comprehensive evaluation of FHAA status and improving patient care.

## Data Availability

The original contributions presented in the study are included in the article/**Supplementary Material**. Further inquires, including request for FHAA ICTs, can be directed to the corresponding author.

## References

[1] Dragon-Durey MA Loirat C Cloarec S Macher MA Blouin J Nivet H. Anti-factor H autoantibodies associated with atypical hemolytic uremic syndrome. J Am Soc Nephrol. (2005) 16:555–63. doi: 10.1681/ASN.2004050380 15590760

[2] Rodríguez De Córdoba S Esparza-Gordillo J Goicoechea De Jorge E Lopez-Trascasa M Sánchez-Corral P. The human complement factor H: Functional roles, genetic variations and disease associations. Mol Immunol. (2004) 41:355–67. doi: 10.1016/j.molimm.2004.02.005 15163532

[3] Durey M-AD Sinha A Togarsimalemath SK Bagga A. Anti-complement-factor H-associated glomerulopathies. Nat Rev Nephrol. (2016) 12:563–78. doi: 10.1038/nrneph.2016.99 27452363

[4] Dragon-Durey MA Sethi SK Bagga A Blanc C Blouin J Ranchin B. Clinical features of anti-factor H autoantibody-associated hemolytic uremic syndrome. J Am Soc Nephrol. (2010) 21:2180–7. doi: 10.1681/ASN.2010030315 PMC3014031 21051740

[5] Hofer J Janecke AR Zimmerhackl LB Riedl M Rosales A Giner T. Complement factor H–related protein 1 deficiency and factor H antibodies in pediatric patients with atypical hemolytic uremic syndrome. Clin J Am Soc Nephrol. (2013) 8:407–15. doi: 10.2215/CJN.01260212 PMC3586960 23243267

[6] Lee JM Park YS Lee JH Park SJ Il SJ Y-H P. Atypical hemolytic uremic syndrome: Korean pediatric series. Pediatr Int. (2015) 57:431–8. doi: 10.1111/ped.12549 25443527

[7] Foltyn Zadura A Zipfel PF Bokarewa MI Sturfelt G Jönsen A Nilsson SC. Factor H autoantibodies and deletion of Complement Factor H-Related protein-1 in rheumatic diseases in comparison to atypical hemolytic uremic syndrome. Arthritis Res Ther. (2012) 14:R185. doi: 10.1186/ar4016 22894814 PMC3580581

[8] Zhang Y Ghiringhelli Borsa N Shao D Dopler A Jones MB Meyer NC. Factor H autoantibodies and complement-mediated diseases. Front Immunol. (2020) 11:607211. doi: 10.3389/fimmu.2020.607211 33384694 PMC7770156

[9] Sinha A Gulati A Saini S Blanc C Gupta A Gurjar BS. Prompt plasma exchanges and immunosuppressive treatment improves the outcomes of anti-factor H autoantibody-associated hemolytic uremic syndrome in children. Kidney Int. (2014) 85:1151–60. doi: 10.1038/ki.2013.373 24088957

[10] Puraswani M Khandelwal P Saini H Saini S Gurjar BS Sinha A. Clinical and immunological profile of anti-factor H antibody associated atypical hemolytic uremic syndrome: A nationwide database. Front Immunol. (2019) 10:1282. doi: 10.3389/fimmu.2019.01282 31231391 PMC6567923

[11] Józsi M Licht C Strobel S Zipfel SLH Richter H Heinen S. Factor H autoantibodies in atypical hemolytic uremic syndrome correlate with CFHR1/CFHR3 deficiency. Blood. (2008) 111:1512–4. doi: 10.1182/blood-2007-09-109876 18006700

[12] Abarrategui-Garrido C Martínez-Barricarte R López-Trascasa M Rodríguez De Córdoba S Sánchez-Corral P. Characterization of complement factor H-related (CFHR) proteins in plasma reveals novel genetic variations of CFHR1 associated with atypical hemolytic uremic syndrome. Blood. (2009) 114:4261–71. doi: 10.1182/blood-2009-05-223834 19745068

[13] Moore I Strain L Pappworth I Kavanagh D Barlow PN Herbert AP. Association of factor H autoantibodies with deletions of CFHR1, CFHR3, CFHR4, and with mutations in CFH, CFI, CD46, and C3 in patients with atypical hemolytic uremic syndrome. Blood. (2010) 115:379–87. doi: 10.1182/blood-2009-05-221549 PMC2829859 19861685

[14] Cugno M Mancuso MC Depetri F Peyvandi F Ardissino G. IgM autoantibodies to complement factor H in C3 glomerulopathy. J Nephrol. (2024) 37:1415–6. doi: 10.1007/s40620-024-01961-4 38824229

[15] Cugno M Berra S Depetri F Tedeschi S Griffini S Grovetti E. IgM autoantibodies to complement factor H in atypical hemolytic uremic syndrome. J Am Soc Nephrol. (2021) 32:1227–35. doi: 10.1681/ASN.2020081224 PMC8259677 33712527

[16] Blanc C Roumenina LT Ashraf Y Hyvärinen S Sethi SK Ranchin B. Overall neutralization of complement factor H by autoantibodies in the acute phase of the autoimmune form of atypical hemolytic uremic syndrome. J Immunol. (2012) 189:3528–37. doi: 10.4049/jimmunol.1200679 22922817

[17] Józsi M Strobel S Dahse HM Liu WS Hoyer PF Oppermann M. Anti-factor H autoantibodies block C-terminal recognition function of factor H in hemolytic uremic syndrome. Blood. (2007) 110:1516–8. doi: 10.1182/blood-2007-02-071472 17495132

[18] Nozal P Bernabéu-Herrero ME Uzonyi B Szilágyi Á Hyvärinen S Prohászka Z. Heterogeneity but individual constancy of epitopes, isotypes and avidity of factor H autoantibodies in atypical hemolytic uremic syndrome. Mol Immunol. (2016) 70:47–55. doi: 10.1016/j.molimm.2015.12.005 26703217

[19] Watson R Lindner S Bordereau P Hunze E-M Tak F Ngo S. Standardisation of the factor H autoantibody assay. Immunobiology. (2014) 219:9–16. doi: 10.1016/j.imbio.2013.06.004 23891327

[20] Parolo C Sena-Torralba A Bergua JF Calucho E Fuentes-Chust C Hu L. Tutorial: design and fabrication of nanoparticle-based lateral-flow immunoassays. Nat Protoc. (2020) 15:3788–816. doi: 10.1038/s41596-020-0357-x 33097926

