# Supplementary material for: Novel immunochromatographic test for rapid detection of anti-factor H autoantibodies with an assessment of its clinical relevance
Source: Front Immunol. 2025 Jan 24;15:1527016. doi: 10.3389/fimmu.2024.1527016 (PMC11802491; doi:10.3389/fimmu.2024.1527016)
Supplement: Supplementary file 1 [file DataSheet1.pdf]

Supplementary Table 1. ICT results in samples that tested negative (AU=0) in the FHAA ELISA

				IMMUNOCHROMATOGRAPHIC DATA (Detection Line)*											
				Batch 1			Batch 2								
DISEASE	Δ CFHR3-CFHR1	SAMPLE	ELISA (AU)	A (AU)	B (AU)	C (AU)	A (AU)	B (AU)	C (AU)	INTERPRETATION IMMUNOCHROMATOGRAPHIC DATA					
aHUS	NO	HUS 1359	0	39	14	83	56	59	61	NEG					
aHUS	NO	HUS 1360	0	33	7	31	50	50	34	NEG					
aHUS	NO	HUS 1364	0	11	15	22	75	42	41	NEG					
aHUS	NO	HUS 1365	0	52	54	78	82	62	116	NEG					
aHUS	NO	HUS 1367	0	2	16	77	72	30	51	NEG					
aHUS	HET	HUS 1368	0	41	39	51	78	73	107	NEG					
aHUS	NO	HUS 1373	0	36	0	33	42	59	16	NEG					
aHUS	NO	HUS 1410	0	41	37	208	77	67	180	Weak POS		IgM			
aHUS	NO	HUS 1413	0	76	0	23	49	38	6	NEG					
aHUS	NO	HUS 1418	0	65	0	28	50	29	70	NEG					
aHUS	HET	HUS 1419	0	39	0	0	43	14	11	NEG					
aHUS	HET	HUS 1420	0	45	0	51	57	20	85	NEG					
aHUS	NO	HUS 1421	0	33	0	13	43	29	27	NEG					
aHUS	NO	HUS 1423	0	48	31	0	45	27	0	NEG					
aHUS	HET	HUS 1424	0	58	0	763	23	0	677	POS		IgM			
aHUS	HOM	HUS 1426	0	78	0	0	76	41	34	NEG					
aHUS	NO	HUS 1427	0	0	0	55	63	50	65	NEG					
aHUS	NO	HUS 1428	0	55	28	21	59	33	66	NEG					
aHUS	HET	HUS 1429	0	0	9	9	63	27	62	NEG					
aHUS	NO	HUS 1430	0	24	15	0	29	42	71	NEG					
aHUS	HET	HUS 1439	0	18	0	62	71	27	81	NEG					
aHUS	NO	HUS 1440	0	28	0	129	57	42	152	Weak POS		IgM			
aHUS	HOM	HUS 191	0	41	0	69	20	67	32	NEG					
aHUS	HOM	HUS 309	0	47	15	28	86	23	51	NEG					
aHUS	NO	HUS 360	0	49	17	98	82	62	78	NEG					
aHUS	NO	HUS 554	0	66	27	17	47	33	34	NEG					
aHUS	HET	HUS 633	0	46	7	38	29	37	27	NEG					
aHUS	HET	HUS 634	0	48	13	2	63	26	0	NEG					
aHUS	HOM	HUS 989	0	116	44	66	73	51	96	NEG					
aHUS	HOM	HUS 854	0	115	0	30	90	41	14	NEG					
aHUS	HOM	HUS 869	0	98	9	102	55	34	60	NEG					
aHUS	HOM	HUS 1052	0	107	41	111	91	51	161	Weak POS		IgM			
aHUS	HOM	HUS 1036	0	22	55	7				NEG					
aHUS	HOM	HUS 1030	0	40	0	40				NEG					

aHUS	HOM	HUS 1108	0	75	52	43				NEG					
aHUS	HOM	HUS 1145	0	78	23	57				NEG					
aHUS	HOM	HUS 1157	0	73	0	37				NEG					
aHUS	HOM	HUS 1172	0	51	51	43				NEG					
aHUS	HOM	HUS 1214	0	65	30	50				NEG					
aHUS	HOM	HUS 1241	0	97	48	55				NEG					
aHUS	HOM	HUS 1256	0	31	57	24				NEG					
aHUS	HOM	HUS 1264	0	60	30	47				NEG					
aHUS	HOM	HUS 1295	0	54	0	38				NEG					
aHUS	HOM	HUS 131	0	68	0	48				NEG					
aHUS	HOM	HUS 132	0	38	49	15				NEG					
aHUS	HOM	HUS 1323	0	55	25	40				NEG					
aHUS	HOM	HUS 1350	0	51	81	29				NEG					
aHUS	HOM	HUS 1356	0	95	0	31				NEG					
aHUS	HOM	HUS 1379	0	60	0	31				NEG					
aHUS	HOM	HUS 146	0	83	37	28				NEG					
aHUS	HOM	HUS 243	0	93	23	34				NEG					
aHUS	HOM	HUS 255	0	98	28	48				NEG					
aHUS	HOM	HUS 266	0	92	20	19				NEG					
aHUS	HOM	HUS 317	0	86	6	4				NEG					
aHUS	HOM	HUS 352	0	72	12	82				NEG					
aHUS	HOM	HUS 367	0	76	29	47				NEG					
aHUS	HOM	HUS 434	0	62	0	32				NEG					
aHUS	HOM	HUS 439	0	82	12	55				NEG					
aHUS	HOM	HUS 445	0	50	44	8				NEG					
aHUS	HOM Δ_{CFHR1}	HUS 499	0	66	31	50				NEG					
aHUS	HOM	HUS 558	0	85	0	50				NEG					
aHUS	HOM	HUS 656	0	76	37	37				NEG					
aHUS	HOM	HUS 719	0	90	53	31				NEG					
aHUS	HOM	HUS 827	0	74	21	16				NEG					
aHUS	HOM	HUS 847	0	49	22	15				NEG					
aHUS	HOM	HUS 079	0	82	25	58				NEG					
aHUS	HOM	HUS 1002	0	65	43	27				NEG					
aHUS	HOM	HUS 887	0	63	6	47				NEG					
aHUS	HOM	HUS 918	0	62	29	0				NEG					
aHUS	HOM	HUS 940	0	47	0	7				NEG					
aHUS	HOM	HUS 964	0	65	31	0				NEG					
aHUS	HOM	HUS 978	0	74	33	51				NEG					
aHUS	HOM	HUS 986	0	41	13	34				NEG					
aHUS	HOM	HUS 1021	0	83	35	39				NEG					
C3G	NO	GN 168	0	95	27	37	102	41	0	NEG					
C3G	HOM	GN 131	0	46	60	248	81	49	370	POS		IgM			

C3G	HOM	GN 194	0	65	64	68	46	60	58	NEG					
C3G	HET	GN 474	0	0	0	120	44	58	65	NEG					
C3G	HET	GN 483	0	53	51	0	61	79	29	NEG					
C3G	NO	GN 490	0	37	58	120	67	55	159	Weak POS		IgM			
C3G	NO	GN 491	0	74	0	161	65	44	127	Weak POS		IgM			
C3G	HET	GN 492	0	30	0	30	48	38	31	NEG					
C3G	HET	GN 493	0	59	0	39	33	21	29	NEG					
C3G	NO	GN 496	0	33	14	243	64	46	215	POS		IgM			
C3G	NO	GN 500	0	582	651	247	724	879	236	POS	IgG	IgM			C-TER
C3G	HOM	GN 459	0	110	16	91	81	26	96	NEG					
C3G	HOM	GN 108	0	81	31	282	80	21	294	POS		IgM			
C3G	HOM	GN 132	0	112	38	58	74	31	103	NEG					
C3G	HOM	GN 154	0	99	52	74				NEG					
C3G	HET	GN 242	0	78	30	18				NEG					
C3G	HOM	GN 272	0	78	41	29				NEG					
C3G	HOM	GN 096	0	64	27	51				NEG					

*) Positive values in the detection band are depicted in red; values above 100AU are considered positive using the Colloidal Gold Rapid Test Strip Reader CHL-TSR100 from Guangzhou Iclear Healthcare Limited. NO, no carrier of $\Delta_{CFHR3\text{-}CFHR1}$. NT, not tested.
